# Multimodality Imaging in the Diagnosis of Coronary Microvascular Disease: An Update

**DOI:** 10.3390/jpm15020075

**Published:** 2025-02-19

**Authors:** Ana Margarida Martins, Miguel Nobre Menezes, Pedro Alves da Silva, Ana G. Almeida

**Affiliations:** 1 Cardiology, Heart and Vessels Department, ULS Santa Maria, Centro Cardiovascular da Universidade de Lisboa, 1649-128 Lisboa, Portugal; miguel.menezes@ulssm.min-saude.pt (M.N.M.); pmsilva@edu.ulisboa.pt (P.A.d.S.); ana.gomes@ulssm.min-saude.pt (A.G.A.); 2 Cardiovacular Magnetic Ressonance Services, Royal Brompton and Harefield Hospitals, 6W3 6NP London, UK; 3 Faculdade de Medicina, Universidade de Lisboa, 1649-028 Lisboa, Portugal

**Keywords:** Coronary microvascular dysfunction, Cardiac imaging, Myocardial ischaemia

## Abstract

Coronary microvascular dysfunction (CMD) is characterized by structural and functional abnormalities in the coronary microvasculature which can lead to ischaemia and angina and is increasingly recognized as a major contributor to adverse cardiovascular outcomes. Despite its clinical importance, the diagnosis of CMD remains limited compared with traditional atherosclerotic coronary artery disease. Furthermore, the historical lack of non-invasive methods for detecting and quantifying CMD has hindered progress in understanding its pathophysiology and clinical implications. This review explores advancements in non-invasive cardiac imaging that have enabled the detection and quantification of CMD. It evaluates the clinical utility, strengths and limitation of these imaging modalities in diagnosing and managing CMD. Having improved our understanding of CMD pathophysiology, cardiac imaging can provide insights into its prognosis and enhance diagnostic accuracy. Continued innovation in imaging technologies is essential for advancing knowledge about CMD, leading to improved cardiovascular outcomes and patient care.

## 1. Introduction

Chest pain is a frequent symptom that commonly leads to coronary angiography due to the suspicion of coronary artery disease (CAD). However, studies indicate that up to 38% of patients undergoing this procedure have normal-appearing coronary arteries [1]. In 1985, Cannon and Epstein introduced the term “microvascular angina” to describe the clinical manifestation of myocardial ischaemia caused by structural or functional changes in the coronary microvasculature [2].

Since then, advancements in both invasive and non-invasive diagnostic techniques have greatly enhanced our understanding of coronary microvascular dysfunction (CMD) and microvascular ischaemia. CMD has become increasingly acknowledged as a major contributor to angina and ischaemia, even in patients with non-obstructive disease of the large or medium coronary arteries; terms such as ANOCA (angina with non-obstructive coronary arteries) and INOCA (ischaemia with non-obstructive coronary arteries) have gained prominence in describing such presentations. However, CMD can also coexist with obstructive epicardial disease, adding further complexity to its diagnosis and management.

CMD can be debilitating and carries an elevated risk of adverse cardiovascular events, such as acute coronary syndrome, heart failure, and sudden cardiac death [3,4]. CMD is also recognized as a key factor in various clinical scenarios, including heart failure and Takotsubo Syndrome [5,6].

Diagnostic testing, whether performed in an interventional cardiology laboratory or through non-invasive imaging techniques, plays a crucial role in confirming the diagnosis of microvascular dysfunction. It also aids in refining risk stratification, guiding therapeutic decisions, and offering objective tools for monitoring the disease. Despite these advances, diagnosing microvascular dysfunction remains challenging, particularly because it can coexist with both obstructive and nonobstructive epicardial disease.

This review aims to present a thorough review of the imaging modalities available for assessing CMD in the absence of obstructive epicardial disease. We will discuss recent advances in diagnostic techniques, their role in risk stratification, and their implications for patient management and treatment outcomes.

## 2. Overview of the Coronary Microvascular Circulation

**Figure 1:**
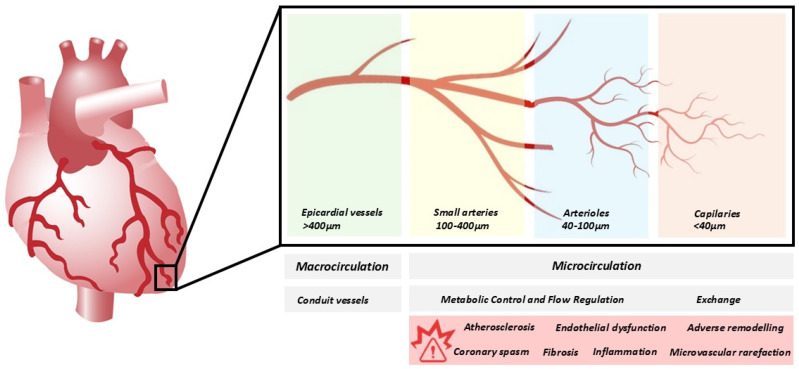
Diagram of Coronary Macro- and Micro-Arterial Systems: Anatomy and Function.

Before exploring the technical aspects of quantifying CMD, a thorough understanding of the functional anatomy of the coronary circulation is crucial. The coronary arterial system is a continuous network composed of functionally distinct vessel segments that progressively decrease in size, each playing a unique role in coronary blood flow regulation [7,8]. The proximal large epicardial coronary arteries, with diameters greater than 400 μm, primarily act as conduits with minimal resistance to blood flow under normal conditions. As these arteries transition into smaller pre-arterioles (100 to 400 μm) and then into intramural arterioles (<100 μm), the system shifts from conductance to resistance. The intramural arterioles make up the majority of the heart’s resistance circuit, crucial for matching myocardial blood supply to oxygen demand by adjusting the coronary blood flow in response to metabolic signals from the myocardium [9] (Figure 1). Myocardial blood flow (MBF) refers to the rate at which blood flows through the coronary arteries; it is commonly measured as the volume of blood delivered per gram of myocardial tissue over a given time period [8].

In a healthy state, the coronary microcirculation relies on complex autoregulatory mechanisms to ensure a relatively constant coronary blood flow despite significant fluctuations in perfusion pressures [8,10]. For instance, during hypotension, reduced driving pressure prompts autoregulatory responses that decrease microvascular resistance, thereby maintaining sufficient blood flow [11]. These dynamic changes stem from a range of mechanisms, including myogenic control, shear-mediated neurohormonal regulation, and metabolic regulation [12].

CMD is a broad term. In the absence of obstructive CAD, the term encompasses any condition that affects the micro-vasculature. This includes endothelial dysfunction, coronary spasm, inflammation, and atherosclerosis, all of which can compromise the autoregulatory mechanisms and impair the ability of the heart to increase the coronary blood flow in response to stress [13]. CMD is marked by an exaggerated response of the coronary microcirculation to vasoconstrictor stimuli and a diminished capacity for microvascular vasodilation. This leads to an insufficient vasodilatory response during exercise or pharmacological stress, which can cause myocardial ischaemia, whether subtle or overt, due to a mismatch between oxygen supply and demand.

Studies have suggested that several cardiovascular risk factors such as smoking, diabetes, hypertension, and dyslipidaemia, are commonly linked to CMD. In a twin study by Rooks et al. [14], smokers exhibited a significantly reduced coronary flow reserve (CFR) (which will be discussed further in this review) compared with nonsmokers. Additionally, evidence suggests a reduced CFR in patients with hyperlipidaemia [15]; there are indications that initiating lipid-reducing treatments may improve coronary flow [16]. In hypertension, extrinsic compression of the microvasculature due to hypertrophy and overactivation of the renin-angiotensin system can contribute to microvascular dysfunction [17].

Chronic inflammation plays a crucial role in the development and progression of coronary atherosclerosis. Recent evidence indicates that systemic inflammatory conditions, such as autoimmune rheumatic diseases, also contribute to the onset of CMD [18]. In patients with systemic inflammatory disorders, impaired coronary vasodilator reserve has been linked to poorer cardiovascular outcomes and an increased all-cause mortality [19]. Additionally, there is growing evidence that psychosocial stress plays a significant role in coronary vasomotor disorders [20].

## 3. Clinical Presentation and Diagnosis

The clinical approach to ischaemic heart disease has traditionally been centred on obstructive CAD, with diagnostic and therapeutic approaches primarily targeting this patient group. Hence, in the absence of obstructive CAD on coronary angiography, an abnormal non-invasive diagnostic test for ischaemia may be misinterpreted as a “false positive.” The advent of advanced diagnostic tools is shifting this paradigm, expanding the definitions of CAD and ischaemia to encompass a broader range of pathological conditions, including coronary microvascular dysfunction.

### 3.1. Clinical Presentation

Patients with CMD frequently present with chest pain that is suggestive of angina and often mirrors the symptoms of obstructive CAD, typically triggered by physical activity and relieved by rest [21,22]. However, atypical presentations such as chest pain occurring at rest, or “angina-equivalent” symptoms such as exertional dyspnoea or fatigue, are also commonly observed in CMD [23]. Patients with CMD may gradually find it harder to tolerate physical activity, often experiencing shortness of breath during exertion. This exertional dyspnoea can indicate ischaemia linked to left ventricular diastolic dysfunction, typically characterized by an elevated pressure response during exercise [24]. It is important to emphasize that the diagnosis of microvascular angina cannot be determined solely based on symptoms.

### 3.2. Assessment of Microvascular Blood Flow

Currently, there is no method available for directly visualizing the coronary microcirculation in vivo in humans. Instead, various measurements that quantify blood flow within the coronary circulation are frequently employed to assess the function of coronary microvasculature [8]. The CFR represents the increase in coronary flow from basal perfusion to maximal vasodilation, reflecting the microvasculature’s ability to respond to stimuli and its functional status. To determine the CFR, myocardial blood flow is measured both at rest and during maximal hyperaemia which is induced by administering adenosine or dipyridamole either intracoronarily or intravenously. The CFR is expressed as the ratio of blood flow during hyperaemia to that at rest; a value below 2.0 is typically considered abnormal. Since CFR values vary by age and sex, comparisons with matched controls are essential. Differences such as smaller vascular diameters and a higher resting coronary blood flow in women likely influence their CFR values, highlighting the need for further research to establish sex-specific cut-offs [25].

Traditionally, the CFR has been applied to the invasive assessment of coronary flow reserve, whereas non-invasive techniques (as explored in this review) address it as the myocardial perfusion reserve (MPR), or the coronary flow velocity reserve (CFVR) in the case of a Doppler assessment. This distinction stems from the fact that non-invasive techniques assess changes in myocardial perfusion rather than directly measuring blood flow at the level of the coronary arteries. The quantification of the MPR by non-invasive methods relies on models that incorporate the temporal dynamics of contrast agents or radiotracers. To reduce the variability in MBF measurements, factors like contrast bolus timing, injection rate, and cardiac output must be carefully considered. The arterial input function (AIF) represents the changing concentration of the radiotracer or contrast agent entering the target tissue over time, accounting for the injection-related variables that influence MBF quantification [26]. Kinetic modeling utilizes the AIF and a compartmental model to quantify the distribution of a tracer or contrast agent in the myocardium. By analyzing concentration-time curves, this method provides a detailed assessment of myocardial tissue function and perfusion dynamics [27].

Transthoracic Doppler echocardiography evaluates the CFVR by calculating the ratio of coronary flow velocity under stress conditions to that at rest [28,29]. This measurement is obtained by sampling the proximal left anterior descending coronary artery using pulsed-wave Doppler.

- Invasive testing

Invasive coronary angiography is a useful approach for assessing patients with CMD as it not only allows for the exclusion of obstructive CAD, but also incorporates catheter-based techniques to evaluate both the epicardial and the microvascular coronary physiology. Recently a standardized protocol has been proposed [30].

Coronary microvascular function is typically assessed invasively by measuring the coronary blood flow response to vasoactive stimuli. This assessment involves two primary techniques: intracoronary thermodilution, which calculates blood flow using a thermal dilution curve, and intracoronary Doppler wire, which measures blood flow velocity through ultrasound based on the Doppler principle [31]. In the absence of large-scale, head-to-head comparisons between these methods, the choice of technique should be guided by the expertise of the centre and the operator. Both techniques allow for the measurement of coronary blood flow velocity both at rest and following the administration of adenosine (for endothelium-independent vasodilation) or acetylcholine (for endothelium-dependent vasodilation) [32]. The thermodilution method allows for the calculation of the index of microvascular resistance (IMR), which is determined by multiplying the mean distal coronary pressure by the mean transit time during maximal hyperaemia. An increased IMR (≥25 U) indicates microvascular dysfunction [31].

The Fractional Flow Reserve (FFR) has become increasingly important in the clinical evaluation of epicardial coronary stenosis, offering a pressure-derived index that provides a surrogate measure of flow limitation and physiological obstruction at the stenosis level. Clinical evidence, particularly from randomized controlled trials like the FAME-2 trial, supports the use of the FFR for guiding revascularization decisions in stable CAD [33]. However, while the FFR is a valuable tool for assessing epicardial conductance, it is not designed to measure coronary microvascular dysfunction. The Instantaneous Wave-Free Ratio (iFR) is a pressure-derived index that assesses stenosis severity without the need for adenosine administration as it is measured during diastole. This trans-stenotic pressure gradient is a reliable indicator of coronary stenosis severity and shows a strong correlation with the FFR [34]. However, like the FFR, the iFR has limitations in the presence of CMD.

- Non invasive techniques

1. Echocardiography

Exercise stress or dobutamine echocardiography is useful for detecting ischaemia in the presence of significant epicardial stenosis, but its role in diagnosing CMD is limited. CMD typically leads to subendocardial ischaemia, which may not produce the clear wall motion abnormalities that are easily visualized in cases with large vessel stenosis. CMD often causes more subtle, patchy ischaemia that may not be detectable through standard qualitative visual assessment. Furthermore, mild hypokinesia can be difficult to interpret consistently, leading to considerable inter-observer variability and reduced diagnostic reproducibility.

The use of echocardiography to diagnose CMD relies on two techniques: myocardial contrast echocardiography and pulsed-wave Doppler sampling of the proximal left anterior descending coronary artery.

The quantification of the MBF using contrast echocardiography was first introduced in 1998 by Wei et al. [35]. This technique involves a continuous venous infusion of air-filled albumin microbubbles containing an inert high molecular weight gas, which remains within the vascular space; it possesses an intravascular rheology similar to that of red blood cells, enabling myocardial opacification and blood flow quantification [36]. The calculation of the MBF relies on both the microvascular cross-sectional area and the mean velocity of the microbubbles. The mean velocity is determined by observing the rate at which microbubbles reappear following their destruction by ultrasound waves, while the cross-sectional area is obtained by measuring the concentration of microbubbles within the myocardium. Vogel et al. validated this approach against Positron-Emission-Tomography (PET), achieving a correlation coefficient of 0.88 when measuring MBFs in healthy volunteers [37]. Although contrast echocardiography has been found effective in evaluating obstructive coronary disease and post–percutaneous coronary intervention microvascular function, its clinical utility in patients with normal coronary arteries and angina symptoms has also been found promising in recent studies [38,39].

**Figure 2:**
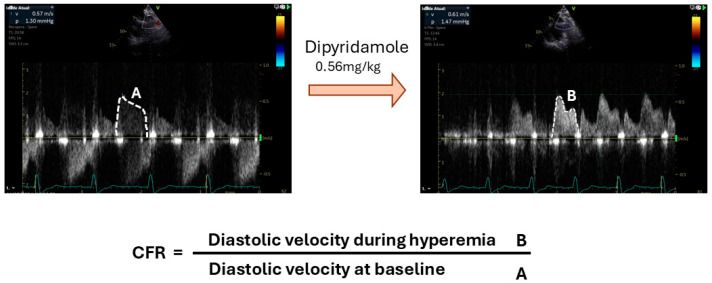
Echocardiography-Derived Coronary Flow Volume Reserve. Using transthoracic Doppler echocardiography with a pulsed-wave sample placed on the anterior descending artery, mean diastolic velocities are measured at rest and during stress to calculate coronary flow velocity reserve.

Another method for assessing the MBF involves using transthoracic Doppler echocardiography to calculate the CFVR [28,29]. The CFVR is determined by measuring the ratio of the coronary flow velocity during stress compared with rest, using a pulsed-wave Doppler to sample the proximal left anterior descending coronary artery (Figure 2). This technique has shown a good correlation with flow measurements obtained from an intracoronary Doppler wire [29,40], and that an abnormal CFVR is linked to an increased risk of adverse cardiovascular events [41]. The technique was also able to identify a high prevalence of CMD in patients with heart failure with preserved ejection fraction in the prospective multicentre international PROMIS-HFpEF study [42]. However, in women with angina and no obstructive CAD, CFVRs measured via transthoracic Doppler echocardiography had a weak correlation (r = 0.30) with the myocardial perfusion reserve (MPR) when assessed by PET [43].

Echocardiographic assessment of CMD is a low-risk, bedside procedure with relatively low cost and minimal adverse effects. Despite these benefits, its adoption has been limited due to several challenges. Echocardiography is highly operator-dependent with significant intraobserver and interobserver variability. Artifacts, especially in patients with obesity or lung disease can further complicate image quality.

2. Cardiac Computed Tomography (CT)

Cardiac CT has recently been increasingly used for functional testing. The combination of CT angiography (CTA) and CT myocardial perfusion imaging (CT-MPI) offers a comprehensive approach for evaluating microvascular disease, as it provides both detailed coronary anatomical information and myocardial perfusion data within a single examination [44]. Two different approaches are available for CT-MPI (static or dynamic).

Static CT-MPI assesses myocardial perfusion by analysing a single dataset acquired during the first-pass contrast enhancement, enabling a qualitative evaluation of regional differences in contrast uptake across the myocardium [45,46]. The assessment of defects is qualitative with hypoenhanced regions compared with normal remote myocardial segments. Similar to nuclear myocardial perfusion imaging, stress images are compared with the baseline perfusion to assess reversibility and to determine myocardial ischaemia. A key limitation of this qualitative approach is its potential to obscure globally reduced myocardial perfusion, as it depends on having a normally perfused region for comparison, and diffuse ischaemia might appear as uniformly low attenuation throughout the myocardium. Additionally, inaccuracies in the bolus timing can lead to missed peak myocardial attenuation, potentially impairing the differentiation between normal and ischemic areas [47].

Dynamic CT-MPI provides a quantitative assessment by capturing multiple sequential CT images following a contrast bolus injection, allowing for the creation of time-attenuation curves that monitor the contrast’s arrival and washout from the myocardium and aorta [48]. It provides diagnostic accuracy comparable to CMR and PET [49]. Through mathematical modelling of these curves, myocardial perfusion can be quantified in absolute terms, offering a more precise evaluation of perfusion dynamics [50]. A MBF below 100 mL/min/100 mL in at least one left ventricular segment indicates microvascular ischaemia. Dynamic CT-MPI combined with CTA has demonstrated a greater sensitivity than static CT-MPI in identifying inducible ischaemia in patients with nonobstructive coronary stenosis detected on angiography [51]. However, dynamic CT-MPI comes with notable limitations, including a higher radiation dose compared with static CT-MPI, and the requirement for prolonged breath-holding which necessitates advanced motion correction algorithms [52,53].

CTA-derived FFR (FFRCT) provides further functional data on the hemodynamic impact of epicardial stenosis by using a three-dimensional anatomical model of the coronariography to apply computational flow dynamics and derive the relative pressures at any point within the coronary circulation. The HeartFlow FFRCT (Redwood City, California) has shown good predictive accuracies when compared with invasive FFR [54,55,56]. The relationship between FFRCT and CMD remains unclear. However, using the same computational modelling, HeartFlow can generate additional parameters that may be valuable for evaluating CMD.

Nørgaard et al. has demonstrated that a low ratio of CTA-derived coronary luminal volume to myocardial mass is an independent predictor of ischaemia in patients with nonobstructive coronary disease [56]. Expanding on this, Grover et al. compared this ratio in 30 patients with microvascular angina, as defined by the European Society of Cardiology guidelines, with 32 age-matched asymptomatic controls [57]. Their findings showed that both coronary luminal volume and myocardial mass were significantly lower in the CMD group, with a notably reduced coronary luminal volume-to-myocardial mass ratio in this cohort.

The key advantage of CTA over other non-invasive methods is its ability to integrate both anatomical and functional assessments of the myocardium and coronary circulation in a single examination. However, although it demonstrates great potential to evaluate microvascular function, a robust validation in this setting is lacking and more prospective research is needed. Further, its routine use for CMD assessment is also limited due to concerns such as radiation exposure and the risk of contrast-induced nephropathy. On this matter, it is worth noting that the exposure of radiation to the foetus can be as high as 5mGy, making it a less useful tool for pregnant patients (especially during the first twelve weeks when the risk of complications rises due to ongoing organogenesis), when comparing with non-invasive methods that are not dependant on ionizing radiation [25,38,58].

3. Nuclear cardiac imaging

PET is the most validated non-invasive imaging modality for identifying CMD [59]. It allows for a comprehensive evaluation, providing global and regional assessments of myocardial perfusion, quantitative MBF, and cardiac function during both stress and rest in a single exam. PET relies on tracers labelled with positron-emitting isotopes that are either actively taken up by myocardial cells or diffuse through the myocardium in proportion to blood flow [12]. Advanced post-processing software is used to automatically segment the heart and measure the arterial input function, enabling precise calculations of both the regional and global MBF at rest and during stress [60,61].

PET radiotracers typically employed in clinical practice include 13N-ammonia, 82Rb, and 15O-water [62]. Among these, 82Rb is the most widely adopted due to its convenience, as it only requires an on-site generator. However, it comes with certain limitations such as a lower extraction fraction, considerable roll-off at higher coronary flows, and an increased radiation exposure. In contrast, 13N-ammonia and 15O-water offer better first-pass uptake, with minimal roll-off. Nevertheless, they are less accessible for routine clinical practice.

PET has been validated and compared with invasive flow estimation methods in several studies, showing a high correlation with an intracoronary Doppler wire (r = 0.82) for the measurement of CFRs [63].

Several prospective PET studies have established a correlation between CMD, defined by an abnormal MPR, and adverse cardiovascular events [64,65]. Ziadi et al., using 82Rb PET imaging, highlighted the prognostic significance of the MPR, showing that it added valuable insights beyond the commonly used summed stress score. Their findings revealed that patients with an MPR below 2, despite having a normal summed stress scores, had more than double the rate of adverse events compared with those with an MPR above 2. Furthermore, they demonstrated that the MPRs, as measured by 82Rb PET, independently predicted serious cardiac outcomes, underscoring the potential benefit of incorporating MBF and MPR assessments into routine clinical evaluations for ischaemia [65].

PET-derived MPRs and MBFs have proven especially valuable for evaluating various patient populations. For example, an abnormal flow reserve in individuals without obstructive CAD has been linked to diastolic dysfunction and a higher risk of hospitalization due to heart failure with preserved ejection fraction [66]. Additionally, patients with metabolic syndrome and type 2 diabetes exhibit impaired MBFs, as measured by PET [11]. These findings are particularly notable in women. Taqueti et al. [67] demonstrated that women often exhibited an impaired flow reserve on PET imaging, despite the absence of obstructive CAD. This impairment was associated with a significantly higher adjusted risk of cardiovascular events (*p* < 0.0001), with a notable interaction effect indicating sex-related differences in risk (*p* = 0.04).

Despite its diagnostic accuracy and the vast amount of prognostic data in PET-derived blood flows, the use of this technique in clinical practice is still limited mainly due its high cost and the risks related to radiation exposure [68].

Early efforts to quantify MBFs using conventional Single-Photon-Emission Computed Tomography (SPECT) imaging yielded mixed results, mainly due to the poor sensitivity and temporal resolution of traditional sodium-iodide cameras [10]. Additionally, commonly used radiotracers, such as technetium-based compounds, show rapid myocardial washout kinetics and substantial liver uptake, further complicating accurate flow measurements [10]. However, the advent of high-sensitivity cardiac cameras has rekindled interest in utilizing dynamic SPECT for MBF quantification. Recently, dedicated cardiac SPECT systems with stationary solid-state cadmium-zinc-telluride (CZT) detectors, offering enhanced sensitivity, spatial resolution, and energy resolution, have been employed for myocardial flow assessments. A prospective study compared dynamic CZT-SPECT with PET and demonstrated its high diagnostic accuracy in detecting reduced MFRs in patients with stable CAD [69].

Despite these advancements, several technical challenges remain that limit the widespread adoption of SPECT for routine quantitative perfusion imaging [70]. While dynamic SPECT protocols for MBF assessments may not match the accuracy and robustness of PET imaging, they hold the potential to provide clinically valuable MFR measurements at a lower cost and in a broader range of facilities that lack access to PET.

4. Cardiac Magnetic Resonance

Cardiac Magnetic Resonance (CMR) is a highly promising non-invasive imaging modality for assessing myocardial perfusion and quantifying blood flow. It offers several advantages, including superior spatial resolution, the absence of ionizing radiation, and excellent diagnostic accuracy, making it a valuable tool in the evaluation of patients with CMD. Two stress CMR approaches have been investigated: stress perfusion and stress T1 mapping.

Currently, the most widely used method for carrying out stress CMR involves inducing hyperaemia with vasodilators like adenosine, regadenoson, or dipyridamole, and administering a gadolinium-based contrast agent (GBCA). Afterwards, serial T1-weighted CMR images are captured to track the GBCA as it passes through the heart chambers and perfuses the myocardium. The qualitative assessment is performed through visual analysis of the first-pass gadolinium images; regions with a normal blood flow show a faster and higher concentration of GBCA leading to a stronger T1-weighted signal, while regions with impaired perfusion exhibit a slower uptake and lower signal intensity.

**Figure 3:**
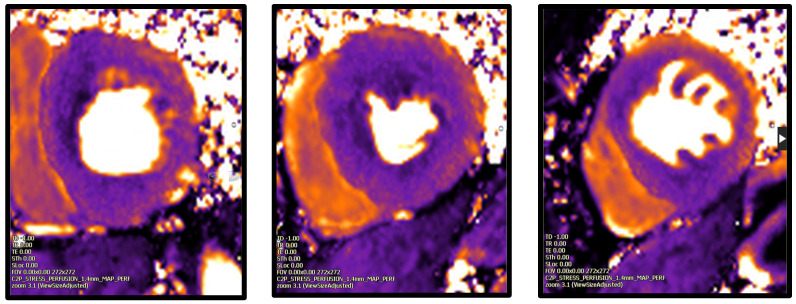
Stress CMR (Cardiovascular Magnetic Resonance) perfusion in a 57-year-old men with hypertension, a history of chest pain, and exertional syncope. The coronary CTA (computer tomography angiography) showed a small calcific plaque with less than 10% stenosis in the proximal left anterior descending artery. On stress first-pass perfusion there was no inducible myocardial perfusion defect. On perfusion mapping (quantitative perfusion) there is an almost circumferential myocardial perfusion defect, with a globally myocardial perfusion reserve (MPR = 1.1) suggestive of microvascular dysfunction. Image kindly provided by the CMR services of Royal Brompton and Harefield Hospitals.

The signal intensity changes observed in first-pass perfusion CMR data can be plotted over time for different regions of interest. The resulting signal intensity/time profiles can then be further analysed by describing the characteristics of the signal intensity changes (‘semi-quantitative analysis’), or by deriving absolute myocardial blood flow values using modelling (‘quantitative analysis’). Fully quantitative perfusion aims to convert a series of CMR images into myocardial blood flow estimates (mL/min/g). This process requires a model that describes the relationship between arterial input and myocardial contrast uptake, often based on the indicator dilution principles adapted for tomographic imaging by Axel [71], and for CMR by Jerosch-Herold et al. [72]. The advantages of quantification include a more objective, less observer-dependent assessment; a simplified analysis; and an improved detection of multi-vessel or microvascular myocardial disease (Figure 3).

Early animal studies evaluating CMR-derived MBFs demonstrated a strong correlation (r > 0.90) with the gold-standard microsphere analysis [73,74]. This success paved the way for several human studies. In patients with stable CAD, global MBF measurements using CMR closely aligned with 13N-ammonia PET (r = 0.92) [75]. Mygind et al. demonstrated a moderate yet significant correlation (r = 0.46, *p* < 0.001) between CMR and PET for identifying microvascular dysfunction in a cohort of women with angina but no obstructive CAD [76]. A potential limitation of CMR-derived MBF assessments has been the lengthy post-processing due to the absence of automated workflows. Kotecha et al. demonstrated that an automated pixel-wise quantitative myocardial perfusion mapping method is effective in distinguishing CMD from three-vessel coronary artery disease with a high level of accuracy [77]. They propose a CMR-based algorithm that utilizes both regional and global stress MBF measurements to identify obstructive CAD and CMD that can be easily implemented into clinical workflows.

Another CMR technique, T1 mapping, has shown promising results for the non-invasive diagnosis of CMD by measuring T1 relaxation time, with no gadolinium contrast. Early data has suggested that patients with type 2 diabetes mellitus and no CAD, display a reduced maximal non-contrast T1 response during adenosine vasodilatory stress compared with controls, likely indicating microvascular dysfunction [78]. In another study, patients with severe aortic stenosis and no obstructive CAD, showed an increased resting myocardial T1 and demonstrated a blunted T1 response to stress. Seven months after AVR myocardial T1 the response to adenosine stress showed improvement [79].

Arterial spin labelling (ASL) is also a promising non-contrast MRI technique that labels the water protons in arterial blood with a magnetic tag, distinguishing them from the surrounding tissue magnetization. This allows for the measurement of blood flow by tracking these “tagged” protons as they circulate [80]. Zun et al. demonstrated that ASL effectively detects clinically significant increases in MBF during vasodilation [81]. These ASL-derived measurements were in agreement with the MBF ranges established by quantitative PET, highlighting its potential as a reliable tool for assessing myocardial perfusion. Currently arterial spin labelling is in the technological developmental stage and holds future promise for the detection of CMD.

Oxygenation-sensitive cardiac magnetic resonance imaging (OS-CMR) is an innovative method for evaluating microvascular dysfunction. It leverages the paramagnetic properties of deoxyhaemoglobin as a natural contrast agent to assess myocardial oxygenation. Paired with standardized breathing manoeuvres, like hyperventilation and apnoea, OS-CMR can detect changes in myocardial oxygenation without the need for intravenous contrast or pharmacologic stress agents. This approach provides valuable markers for both coronary macro- and microvascular function, uniquely capturing rapid, dynamic shifts in oxygenation in vivo and offering significant diagnostic potential for coronary vascular health [82].

In a recent study, Zhou W. et al. explored the prognostic value of assessing CMD using CMR in 218 patients with angina but without obstructive CAD [83]. They found that the stress perfusion CMR-derived myocardial perfusion reserve index is a significant independent marker for predicting MACE in this patient population, highlighting its utility in assessing risk over the medium term.

Unlike other techniques, CMR provides the unique advantage of simultaneously assessing myocardial perfusion and structural abnormalities, including tissue characterization. This distinct feature is particularly valuable in specific pathologies such as aortic stenosis and hypertrophic cardiomyopathy, where it enables the evaluation of both the extent of hypertrophy and the presence of myocardial fibrosis [84].

Despite its limitations, CMR offers a significant advantage since it involves no radiation exposure. In pregnant patients, magnetic radiation has no deleterious effects on the foetus; however, it should be noted that most guidelines recommend against the use of gadolinium-based contrast agents in pregnant women, as it may increase the risk for rheumatological and inflammatory conditions in the newborn. Despite concerns that gadolinium can reach infants through lactation, its water solubility limits excretion in breast milk and there are no reports of harm; thus, lactation should not be interrupted after an MRI exam with gadolinium [25,85]. Furthermore, continuous technological advancements are expected to reduce examination times, enhance patient comfort, and potentially lower costs.

## 4. Treatment

The key objective in identifying CMD is to establish prognostic implications and tailor treatment approaches. However, the multifactorial pathophysiology and overlapping phenotypes make CMD a particularly challenging condition to manage. The outcomes of existing therapeutic trials have been constrained by inconsistent patient selection, as well as inadequate study designs characterized by small sample sizes and a lack of clear evidence of clinical improvement [13].

Given the strong association between cardiovascular risk factors, atherosclerosis, and CMD, individualized lifestyle guidance is important to manage risk factors, moderate symptoms, and improve both quality of life and long-term outcomes. This involves smoking cessation, weight loss, strict management of blood pressure and diabetes, along with lipid control, better diet, and regular physical activity. Optimized medical therapy has demonstrated significant reductions in myocardial ischaemia in patients with stable CAD [86], often exceeding the benefits seen with atherosclerotic plaque regression [87], likely due to improved function of the coronary microcirculation. Furthermore, surgical weight loss has been linked to improved microvascular function, likely driven by better management of cardiovascular risk factors [88].

Symptomatic management of patients with microvascular angina is often difficult due to their heterogeneity and the lack of large, randomized trials. A small study demonstrated that a stratified antianginal therapy approach based on coronary functional testing, improved angina symptoms and quality of life compared with standard therapy [89]. Beta-blockers, calcium channel blockers, ranolazine, and angiotensin-converting enzyme inhibitors are commonly used and can relieve symptoms of microvascular angina [90]. Notably, anti-ischaemic therapies like amlodipine or ranolazine have shown significant improvements in exercise tolerance in these patients [91]. When coronary vasospasm is believed to be the main underlying mechanism, calcium antagonists should be considered as first-line therapy. Nitrates should be considered to prevent recurrent episodes.

Several ongoing studies are currently investigating therapies tailored specifically for patients with CMD. The Women’s a Trial to Reduce Events in Non-ObstructIve CORonary Artery Disease (WARRIOR, NCT03417388) [92] is a multicentre, prospective, randomized trial assessing whether intensive statin and ACE-I/ARB therapy (ischaemia-focused medical treatment) can reduce MACE in symptomatic women with signs of ischaemia and no obstructive coronary artery disease, compared with standard care. Another promising trial, the Precision Medicine with Zibotentan in Microvascular Angina (PRIZE, NCT04097314) [93], is exploring the potential of Zibotentan, an oral endothelin A receptor antagonist, to alleviate microvascular angina by counteracting the vasoconstrictive effects of endothelin on coronary micro vessels.

## 5. Conclusions

CMD poses significant diagnostic and therapeutic challenges, but recent advancements in non-invasive imaging techniques offer promising solutions (Table 1 summarizes the strengths and weaknesses of non-invasive imaging modalities for CMD diagnosis). Various imaging modalities, including PET, CMR, and CT, are now available to measure MBFs and MPRs, key markers for diagnosing CMD. While PET remains the most validated technique with extensive clinical and prognostic data, CMR-derived MBFs and T1-mapping measures are being increasingly used to diagnose patients and to assess their response to treatment.

Limitations remain, particularly the inability of current stressors (such as adenosine and dipyridamole) to assess endothelial function or coronary spasms, and the necessity for ruling out obstructive CAD before diagnosing CMD. Hybrid imaging approaches like CCTA with perfusion, and PET-CT, offer a more comprehensive evaluation by combining anatomical and functional assessments in a single test.

Future research should focus on further validating these techniques, refining algorithms for CMD diagnosis, and developing integrative imaging approaches. Recognizing CMD is crucial for its prognostic significance, and continued advancements in non-invasive cardiac imaging, will be essential for improving the identification, treatment, and management of this condition, ultimately addressing the substantial morbidity and mortality associated with CMD.

## Figures and Tables

**Table 1:** Advantages and drawbacks of commonly used non-invasive modalities for diagnosing CMD.

Imaging Modality	Advantages	Drawbacks	Sensitivity/Specificity
Echocardiography	Easy access Low risk Low cost	Significant intraobserver and interobserver variability. Artifacts. Acoustic window limitations	+/++ [94]
Cardiac CT	Anatomic and functional data in the same study	Potential nephrotoxicity Exposure to radiation Potential for overestimating MBF	++/+++ [84]
PET	Most extensively validated technique Strong prognostic value High accuracy and reproducibility Not constrained by renal function	High cost Radiation exposure Limited accessibility Time-consuming process	+++/+++ [95]
SPECT	More widely available than CMR or PET	Requires new generation cameras Radiation exposure Limited data	+/++ [73,74]
CMR	High spatial resolution and tissue characterization No radiation exposure Validated and compared with PET and invasive techniques	High costs Limited by renal function Limited accessibility Time consuming	+++/++ [77,84]

+ (low): Sensitivity or specificity between **50–64%**; ++ (moderate): Sensitivity or specificity between **65–80%**; +++ (high): Sensitivity or specificity **≥90%**.

## Abbreviations

AIF	arterial input function
ASL	arterial spin labelling
CAD	coronary artery disease
CFR	coronary flow reserve
CFVR	coronary flow velocity reserve
CMD	coronary microvascular dysfunction
CMR	cardiac magnetic resonance
CT	computed tomography
CTA	computed tomography angiography
CT-MPI	computed tomography myocardial perfusion imaging
CZT	cadmium-zinc-telluride
FFR	fractional flow reserve
FFRCT	computed tomography angiography derived fractional flow reserve
GBCA	gadolinium-based contrast age
iFR	instantaneous wave-free ratio
IMR	index of microvascular resistance
MACE	major adverse cardiovascular events
MBF	myocardial blood flow
MPR	myocardial perfusion reserve
OS-CMR	oxygenation-sensitive cardiac magnetic resonance
PET	Positron Emission Tomography
SPECT	Single-Photon-Emission Computed Tomography
